# Structural Basis for Xenon Inhibition in a Cationic Pentameric Ligand-Gated Ion Channel

**DOI:** 10.1371/journal.pone.0149795

**Published:** 2016-02-24

**Authors:** Ludovic Sauguet, Zeineb Fourati, Thierry Prangé, Marc Delarue, Nathalie Colloc'h

**Affiliations:** 1 Unité de Dynamique Structurale des Macromolécules (UMR 3528 CNRS) Institut Pasteur, Paris, France; 2 Laboratoire de cristallographie et RMN biologiques (UMR 8015 CNRS), Paris, France; 3 CNRS, UMR 6301, ISTCT CERVOxy group, GIP Cyceron, Caen, France; 4 UNICAEN, Normandie Univ., UMR 6301 ISTCT, Caen, France; 5 CEA, DSV/I2BM, UMR 6301 ISTCT, Caen, France; Zhejiang University, CHINA

## Abstract

GLIC receptor is a bacterial pentameric ligand-gated ion channel whose action is inhibited by xenon. Xenon has been used in clinical practice as a potent gaseous anaesthetic for decades, but the molecular mechanism of interactions with its integral membrane receptor targets remains poorly understood. Here we characterize by X-ray crystallography the xenon-binding sites within both the open and “locally-closed” (inactive) conformations of GLIC. Major binding sites of xenon, which differ between the two conformations, were identified in three distinct regions that all belong to the trans-membrane domain of GLIC: 1) in an intra-subunit cavity, 2) at the interface between adjacent subunits, and 3) in the pore. The pore site is unique to the locally-closed form where the binding of xenon effectively seals the channel. A putative mechanism of the inhibition of GLIC by xenon is proposed, which might be extended to other pentameric cationic ligand-gated ion channels.

## Introduction

Gaseous anesthetics like xenon (Xe) and nitrous oxide (N_2_O) have been used in clinical practice for decades. Xenon, whose general anesthetic properties were discovered in 1951 [1] has been widely used in anesthesia since mid-2000 despite its excessive cost [2–4]. The main interest of xenon resides in its remarkably safe clinical profile with a rapid pulmonary uptake and elimination, no hepatic or renal metabolism. It readily crosses the blood brain barrier and has a low solubility in blood, which is advantageous in terms of rapid inflow and washout [2, 4, 5]. In addition, xenon has been shown to be a very promising neuroprotective agent in ischemic stroke [6–9], neonatal asphyxia [10, 11], and traumatic brain injury [12]. Xenon targets several neuronal receptors, such as the N-methyl-D-aspartate (NMDA) glutamatergic receptor [13] and the TREK-1 two-pore domain K^+^ channel [14]. In addition, xenon alters neuronal excitability by modulating agonist responses of cationic pentameric ligand-gated ion channels (pLGICs). Indeed, xenon inhibits the excitatory cationic nicotinic acetyl-choline (nAChR) receptor [15, 16] while it has a minimal effect on inhibitory anionic γ-amino-butyric type-A receptor (GABA_A_R) [17–20].

The mechanisms by which noble gases like xenon interact with proteins have been investigated by protein X-ray crystallography under pressurized gas [21–24] or ^129^Xe NMR spectroscopy [25, 26]. These structural studies allowed the characterization of the gas-binding properties and improve the understanding of how chemically and metabolically inert gases produce their pharmacological action. Computational studies on gas/protein interactions [27–32] confirmed that xenon binds within hydrophobic cavities through weak but specific induced dipole-induced dipole interactions [21, 33].

However, up to now all X-ray crystallographic studies were performed solely on globular proteins as surrogate models for physiological neuronal targets [34–37]. Very few structural studies have been performed on xenon interactions with neuronal ion channels. For example xenon binding sites in NMDA receptor were studied only by molecular modeling, which concluded that xenon would be a competitive inhibitor of glycine to its binding site [38–40].

To improve the understanding of molecular interactions between xenon and transmembrane receptor targets, we investigated xenon binding with the *Gloebacter violaceus* ligand-gated ion channel (GLIC), a member of the pLGIC family, using X-ray crystallography under pressurized gas. Previously, the sensitivity of GLIC to gaseous anesthetics has been studied using 2-electrode voltage clamping techniques [41] and this revealed that GLIC currents are inhibited by clinical concentrations of xenon.

In vertebrates, the pLGIC family splits into the cation-selective serotonin and nACh receptors on one hand, and the anion-selective GABA and glycine receptors on the other hand [42]. GLIC, whose X-ray structures has been solved in open [43, 44], locally-closed (LC)–inactive- [45] and resting-state conformations [46], is extensively used to identify binding sites of general anesthetics [47], channel blockers [48], alcohols [49], and other allosteric modulators in pLGICs [50]. Here, we show by X-ray diffraction that xenon has multiple and specific binding sites in GLIC either in the open and LC (inactive) conformations, and that these binding sites differ between the two conformations. A putative mechanism of inhibition of GLIC by xenon can then be proposed, based on the observed xenon binding sites in both forms.

## Materials and Methods

### Crystallization

The GLIC receptor was expressed as a fusion to MBP and purified as described previously [45]. All crystallization experiments were made using vapour diffusion in hanging drops at 18°C as described previously [51]. While GLIC open form crystals were obtained from the GLIC wild-type receptor, the GLIC LC form crystals were obtained from the GLIC C27S-K33C-K248C triple-mutant. This mutant displays a disulphide-bridge between the Loop 2 and the M2-M3 loop that was shown to trap the receptor in its LC-form [45].

### Xenon pressurisation and diffraction data recording

Crystals of both forms were fished out from drops using Hampton® cryoloops and were pressurized in a XCell® from Oxford Cryosystems at a standard pressure of 2 MPa of xenon. After about 5 min, the pressure was released and the loop immediately plunged in liquid nitrogen for quenching. It was known from earlier studies that GLIC crystals do not diffract equally, even coming out from the same drop of crystallization. We thus selected about ten crystals of each form to be exposed to xenon according to the above-described protocol. The best diffracting crystal was selected for diffraction recordings by analysing a single test frame.

Data recordings were conducted at the ESRF synchrotron (Grenoble, France) for the open form of GLIC (beam line BM30A) and at SOLEIL synchrotron (Gif-sur-Yvette, France) for the LC form of GLIC (beam line PROXIMA-1). Wavelengths were chosen within the range 1.1–1.3 Å as a compromise between a significant anomalous signal for xenon and the deleterious effects of absorption. The two data sets of frames were processed using XDS [52]. The resulting independent structure factor amplitudes were formatted using the CCP4 package of programs [53]. The different parameters and statistics of the recording conditions are reported in the Table 1.

**Table 1:** Data collection and refinement statistics for Xenon bound to GLIC in open and LC conformations.

	Xe + GLIC open	Xe + GLIC LC
*Data collection parameters*		
Wavelength (Å)	1.1820	1.200
Δf”	4.6	4.8
Space group	C2	C2
Cell dimension a, b, c (Å)	180.01, 132.82, 159.16	176.99, 130.53, 157.53
β (°)	101.88	100.33
Resolution range (Å)	48.7–3.1 (3.27–3.10)	48.0–3.4 (3.60–3.40)
Number of unique reflections	66304 (9639)	47834 (6881)
Redundancy	3.7 (3.8)	3.0 (3.0)
Completeness (%)	99.6 (99.9)	98.4 (97.1)
I/sigma (I)	9.9 (2.0)	12.0 (1.8)
R_merge_ (%)	9.2 (66.5)	5.2 (58.6)
*Refinements*		
Resolution range (Å)	25.0–3.10	20.0–3.40
R factor / Free R factor (%)	20.9/22.8	21.6/22.3
*Number of atoms*		
Protein	12677	12604
Water	7	-
Lipids	370	-
Ions	51	34
Detergent	72	12
Xenon	30	21
*B factor (Å*^*2*^*)*		
Protein	87.6	127.6
Water	37.3	-
Lipids	109.7	-
Ions	94.4	116.9
Detergent	71.1	105.9
Xenon	79.5	102.6
*R*.*m*.*s*. *deviations*		
Bond length (N° / value in Å)	13572 / 0.008	12885 / 0.008
Bond angles (N° / value in °)	18445 / 0.98	17624 / 1.03
Peptide Omega torsion angle (N° / value in °)	1550 / 2.40	1550 / 2.78
Estimated coordinate error (Å)*	0.650	0.884
*Ramachandran*		
Preferred (%)	95.9	97.8
Outliers (%)	0	0
Molprobity scores #	99^th^	100^th^

* From Luzzati plots

# 100^th^ percentile is the best among structures of comparable resolution

Values in parenthesis are for the highest resolution shell

### Structure refinement

The xenon-bound GLIC open and LC structure were solved by molecular replacement using, as starting models, the structure of *apo* GLIC open (PDB 4HFI) and *apo* GLIC C27S-K33C-L246C triple-mutant (PDB 3TLV), respectively. The resulting model refinement was performed using *autoBUSTER* [54]. Automatically-generated non-crystallographic symmetry restraints were used throughout refinement. MolProbity scores for the refined xenon-bound GLIC open and LC models respectively ranged within the 99^th^ and 100^th^ percentiles of structures refined at comparable resolutions. Refinement statistics are summarized in Table 1. Strong Fo-Fc and anomalous electron density peaks indicated the presence of xenon in both structures. The occupancies of xenon sites were adjusted in such a way that, after refinement, their B-factors matches those of the neighbouring protein atoms. The list of the xenon sites is reported in Table 2. Lipids, ions and detergents occupying positions that were already characterized in a previous work [51], were also added to the model, when appropriate. Coordinates and structure factors of the GLIC open and GLIC LC xenon-bound X-ray structures are deposited under the PDB codes 4ZZC and 4ZZB, respectively.

**Table 2:** Xenon sites in GLIC based on the height of their anomalous signal (given in sigma's above the mean electron density of the maps). The height of the Fo-Fc map is also given. The error is calculated by comparing the same site in the five different subunits of the pentamer.

	Anomalous peaks (in σ)	Fo-Fc difference peaks (in σ)
**GLIC open**		
*Major sites*		
Intra-subunit sites	9.84 ± 1.62	20.20 ± 1.45
Inner-interfacial sites	10.20 ± 1.52	20.50 ± 1.48
*Minor sites*		
Extra-cellular sites	3.92 ± 0.43	3.96 ± 1.62
Minor-interfacial sites	4.08 ± 0.25	6.74 ± 1.05
Membrane-exposed sites	2.76 ± 0.34	5.46 ± 0.42
**GLIC LC**		
*Major sites*		
Intra-subunit sites	7.31 ± 1.06	10.1 ± 0.63
Outer-interfacial sites	14.98 ± 1.62	10.3 ± 0.62
Pore site	7.90	10.10
*Minor sites*		
Extra-cellular sites	5.82 ± 0.68	7.92 ± 0.63
Membrane-exposed sites	3.88 ± 0.62	4.81 ± 0.43

## Results

### Xenon-binding sites in GLIC open and LC forms

**Fig 1:**
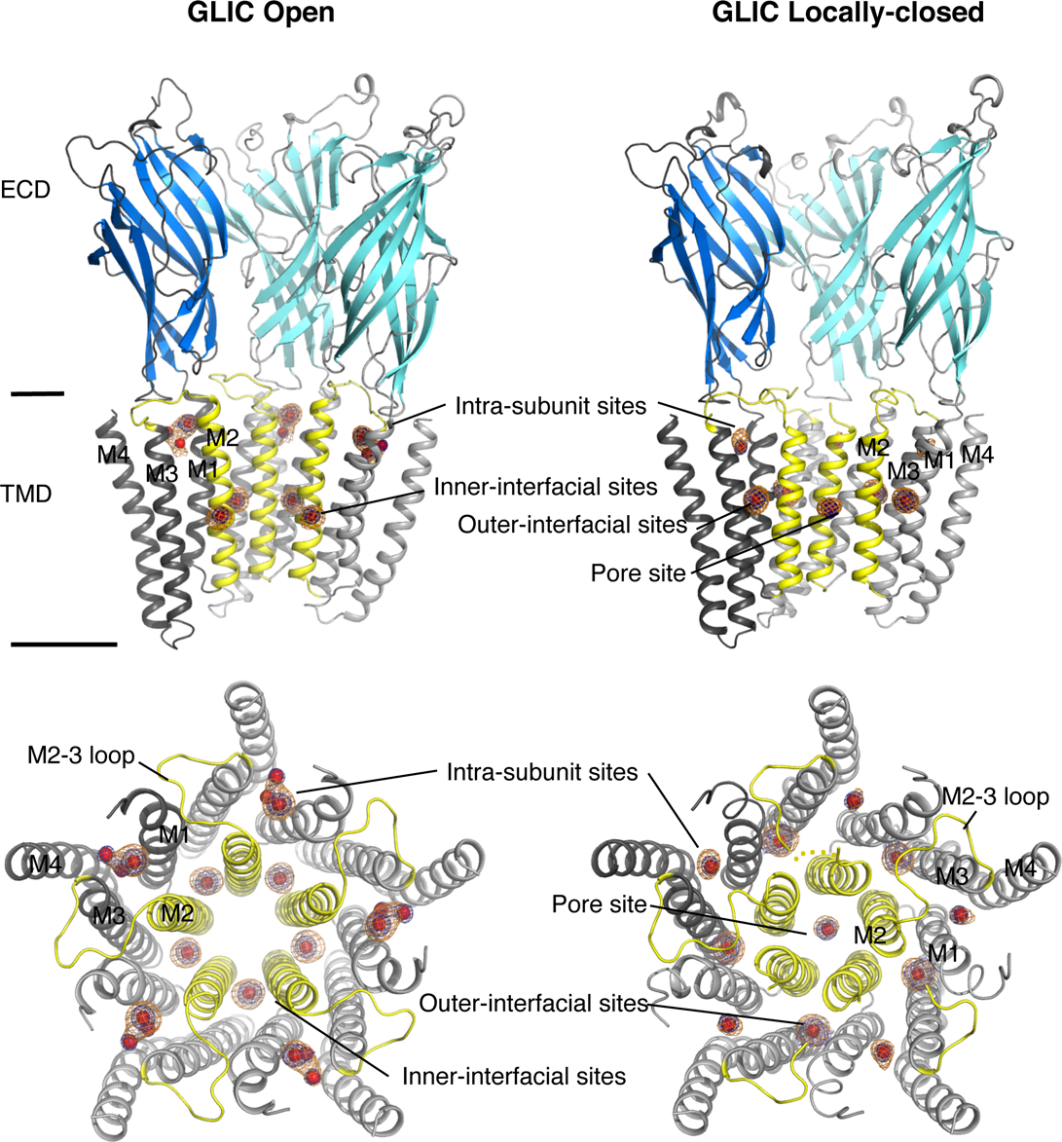
Major xenon-binding sites in GLIC open (left) and LC conformations (right). Top panels: the receptors are viewed from the side with the two front subunits removed to allow a better visualization of the Xenon-binding sites. Bottom panels: the TMD are viewed from the top. Regions of the TMD that differs between the open and LC GLIC conformations are highlighted in yellow, other regions of the TMD are coloured in grey; the ECD is coloured in cyan. The anomalous (orange) and 2mFo-dFc averaged Fourier difference (blue) maps surrounding Xenon binding sites are shown by mesh contoured at 5.0 σ and 10.0 σ, respectively. Xenon atoms are shown by red van der Waals spheres.

Xenon-bound GLIC open and LC structures were obtained from crystals pressurized with 2 MPa (20 bar) of xenon and analysed by X-ray crystallography at 3.1 and 3.4 Å resolution respectively (Table 1). No pressure is required to reveal *in-vivo* anesthetic effects but due to slow gas penetration in crystalline systems, it is admitted that a 10-fold higher pressure is usually required to allow gas binding saturation in the crystals [55]. Both xenon-bound structures are superimposable with their *apo* forms, except for strong Fo-Fc electron density peaks indicating the presence of xenon in distinct cavities. X-ray data were collected at an optimized wavelength of 1.1–1.3 Å in order to maximize the xenon anomalous signal and confidently assign presence of xenon in these electron density peaks (Table 2). Xenon-binding sites are distributed from the ECD (*Extra Cellular Domain*) to the TMD (*Trans-Membrane Domain*) and are located either within subunits of the receptor or at the interface between adjacent subunits (Fig 1 and S1 Fig). Xenon-binding sites are symmetrically distributed in all five subunits except for the pore binding site that is located along the non-crystallographic 5-fold symmetry axis of the pentamer in the LC form (Fig 1). Xenon-binding sites were split into two groups, major or minor sites, depending on their occupancies. Major sites were refined with occupancies ranging from 0.7 to 1.0, and they were initially associated with peaks 7.3 to 15.0 σ in anomalous *F±* maps, and with peaks ranging from 10.1 to 20.2 σ in *Fo-Fc* Fourier difference maps. Minor sites were refined with occupancies ranging from 0.2 to 0.4 and were associated with anomalous peaks and *Fo-Fc* Fourier difference peaks ranging from 2.8 to 5.8 σ, and from 3.9 to 7.9 σ respectively (Table 2). Hereafter, we will mainly focus on the major xenon-binding sites that occupy functionally relevant locations in the receptor that are all reshaped within the GLIC open and LC forms. The minor xenon-binding sites will be briefly described.

### Major xenon-binding sites cover both known and novel modulation sites

Xenon major binding sites are clustered in three distinct regions of the TMD: in a large intra-subunit cavity, in the pore, and at the interface between adjacent subunits (Fig 1).

#### 1. Xenon occupies an intra-subunit general anesthetic (GA) binding cavity

**Fig 2:**
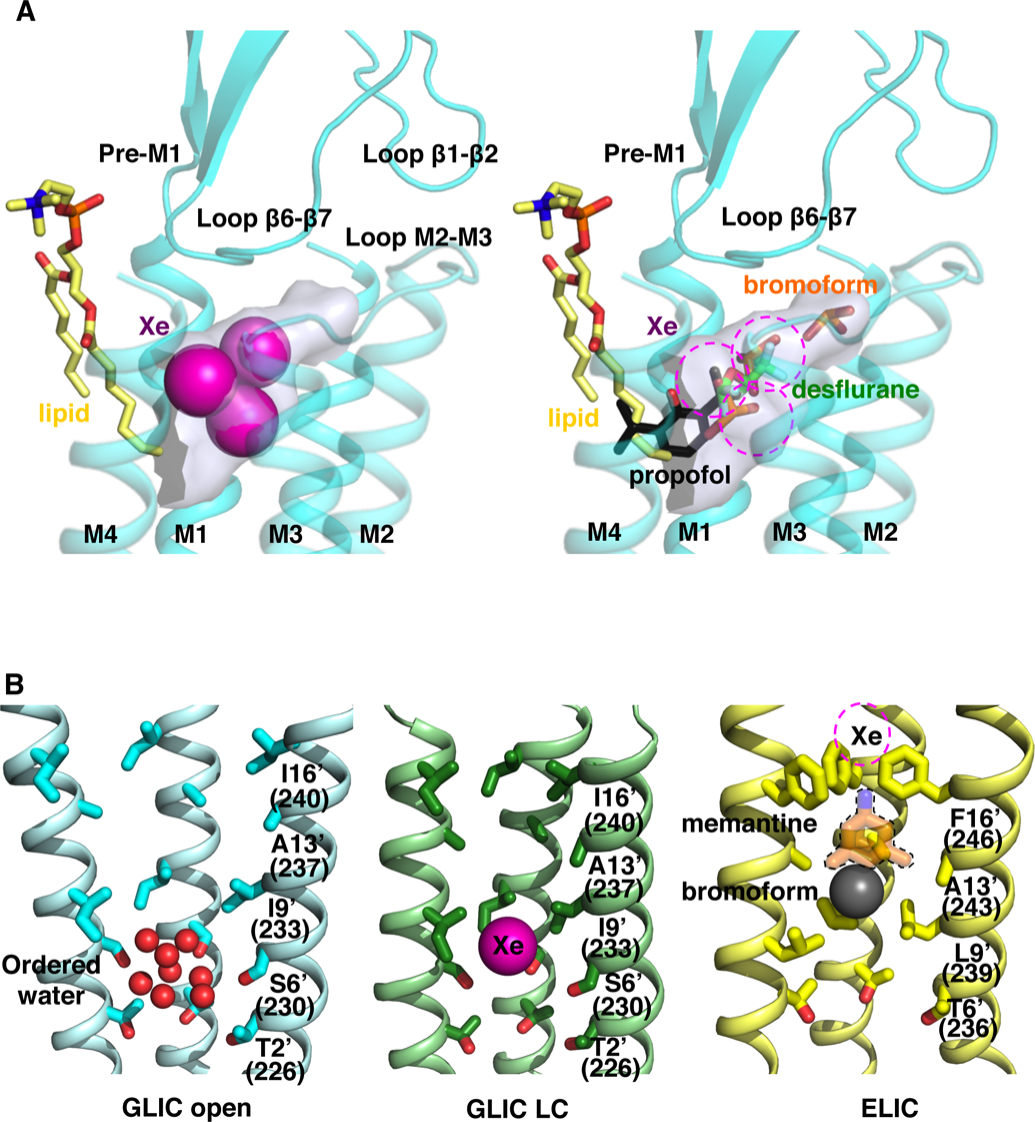
Xenon-binding sites in GLIC overlap with other modulators binding sites. (A): Intra-subunit xenon-binding sites (left panel) overlap with the binding sites of propofol (3P50), desflurane (3P4W) and bromoform (4HFH) (right panel) in GLIC open form. (B): The pore xenon-binding site observed in GLIC LC (middle panel) matches a region that is critical both for ion transport and gating in GLIC open form. Ordered water molecules are observed at this location in GLIC open form (left panel). Same view of the pore in ELIC closed structure (right panel) showing a superimposition of the binding sites observed for memantine (4TWD) and bromoform (3ZKR). The limited resolution of the bromoform-bound ELIC structure did not allow to assign a unique binding pose for bromoform [57]. Bromoform was thus modelled as a single bromine atom, shown as a sphere.

**Fig 3:**
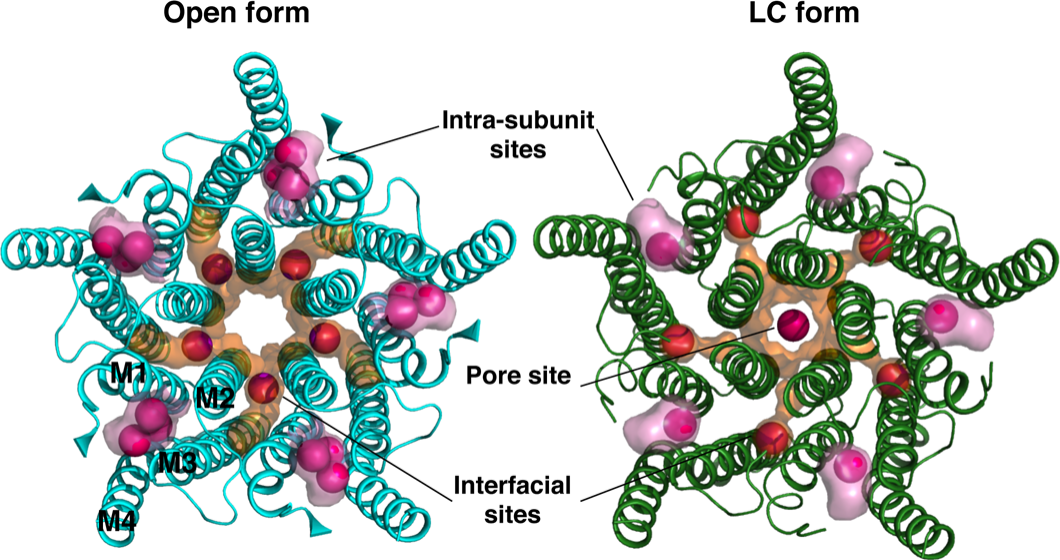
Remodelling of transmembrane major xenon-binding cavities in GLIC open and LC conformations. Cartoon representations of the GLIC TMD viewed from the top in its open (blue) and LC (green) conformations. Xenon-binding cavities in GLIC are delimited by a transparent surface and xenon atoms are illustrated by van der Waals spheres (magenta).

**Fig 4:**
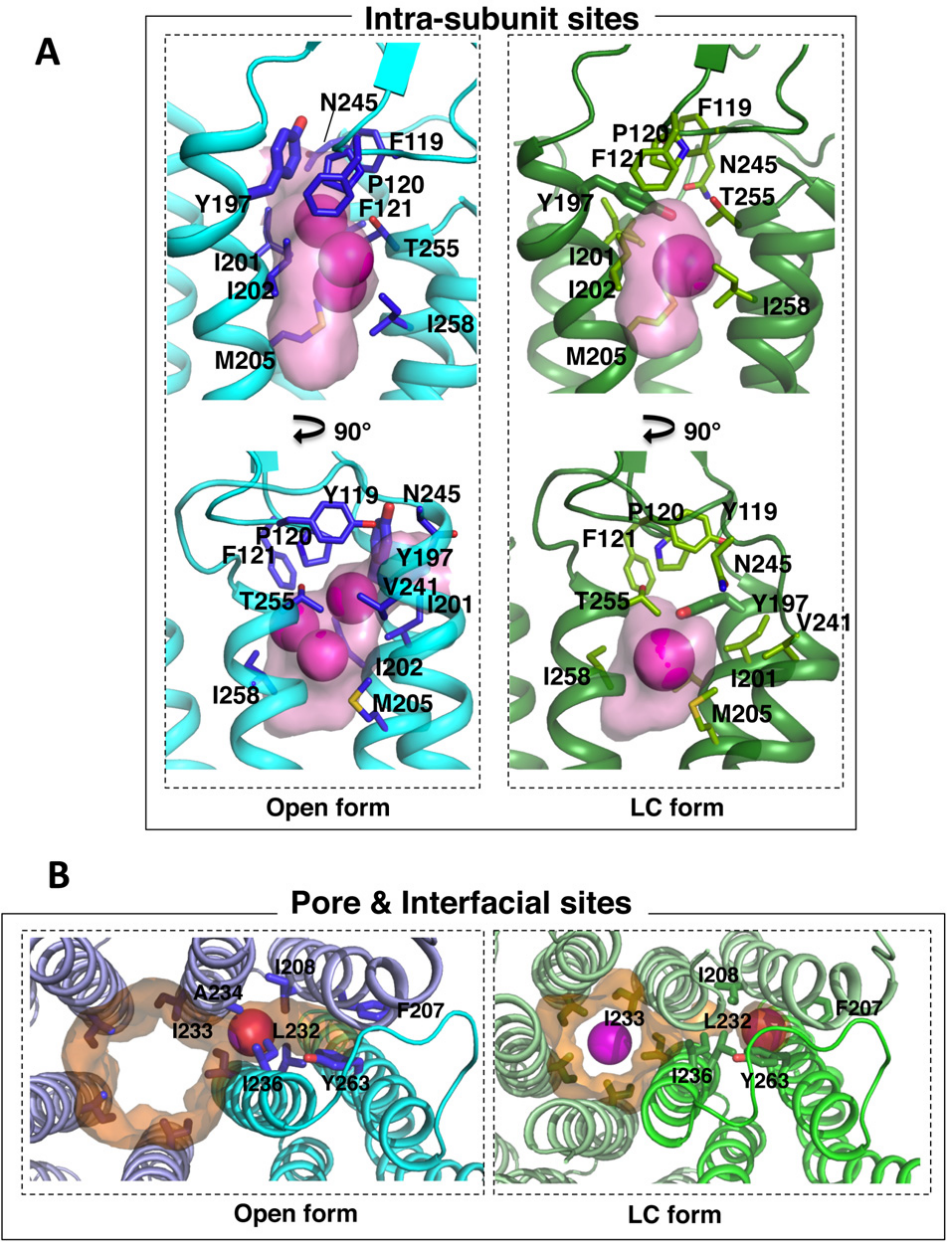
Transmembrane xenon-binding cavities in GLIC open and LC conformations. Enlarged views of the intra-subunit sites (A), the interfacial and pore sites (B) observed upon remodelling of transmembrane xenon-binding sites. Colour code is the same as in Fig 3.

In GLIC open form, xenon is found at three close but different locations in an intra-subunit cavity located in the upper half of the TMD (Fig 2A). This cavity is lined by residues from a single subunit with contributions from helices M1 (Y197, I201, I202 and M205), M2 (V242), M3 (Y254, T255, I258 and I259), M4, the apex of the β6- β7 loop (Y119, P120, F121) and the M2-3 loop (N245) (Fig 3 and Fig 4A).

The cavity is accessible from the lipid bilayer, it penetrates the interior of the subunit along a direction that points towards the ECD-TMD interface. The cavity is 8 Å large at its entrance but progressively narrows down to 4 Å. Additional nearby electron density can be accounted for by a lipid molecule whose alkyl chains border the cavity entrance (Fig 2). The first two locations of xenon are observed at the pocket entrance and were modelled as alternative poses of the same xenon atom with 0.5 occupancy each. The third xenon atom with 0.9 occupancy is deeply buried within the cavity in a highly hydrophobic environment. The xenon atoms are all engaged in van der Waals interactions with the hydrophobic side chains of surrounding residues and the alkyl chains of the lipid molecule (Fig 2A and Fig 4A). Earlier studies revealed that structurally diverse general anesthetics (GAs) like propofol, desflurane [47] and bromoform [49] all bind to this cavity in GLIC open form and partly overlap with the xenon intra-subunit sites (Fig 4A). This present study confirms the earlier observation of GAs-binding to this cavity and extends it to the first example of a gaseous anaesthetic.

Comparing the xenon-bound GLIC open and LC structures shows that this intra-subunit cavity is markedly reshaped upon channel closure (Fig 3 and Fig 4A). In the GLIC LC form, the bottom end of the cavity is obstructed because of a revolving motion of helix M2 and the M2-3 loop, and a conformational flip of the Y197 side chain (Fig 4A). The phenol ring of Y197 points toward the ECD/TMD interface in GLIC open form (upward conformation) and interacts through hydrogen bonding with the hydroxyl moiety of Y119 from the β6-β7 loop and with the ammonium moiety of K248 from the M2-3 loop. Upon channel closure, the revolving motion of the M2-3 loop destabilises this network of interactions and Y197 predominantly adopts a downward conformation stabilized through hydrogen bonding with T255 (M3 helix). In three out of five subunits the side chains of Y197 is found to occupy both the upward and downward conformations suggesting it regularly switches from one position to the other under physiological conditions. This local conformational change has already been observed for other GLIC crystal structures solved in the LC form [45]. Interestingly, the downward conformation of Y197 partially occludes the intra-subunit cavity in the GLIC LC form and is likely to compete with xenon binding. Indeed, the xenon-bound GLIC LC structure reveals that the contraction of the intra-subunit cavity constrains xenon binding to one single location at the cavity entrance. This unique xenon binding site has a markedly lower occupation (0.7) than the xenon intra-subunit sites in GLIC open form (0.9–1.0) further illustrating that the reshaping of the intra-subunit cavity in GLIC LC form limits xenon-binding to this cavity. In addition, the fact that the phospholipid located at the entrance of the cavity is destabilized in the GLIC LC form [45] might also contribute to prevent optimal binding of xenon in this position.

#### 2. Xenon specifically binds within the pore of the GLIC LC form

Xenon binds to a novel site located in the pore, in (and only in) GLIC LC form (Fig 2B). This site is located in the middle of the channel pore, on the receptor five-fold symmetry axis, between the S230 and I233 rings of residues (respectively S6’ and I9’ in a prime notation that starts from the N-terminus of M2 helix). Interestingly, this site is specific to the GLIC LC form as no xenon binds in the pore in the GLIC open form. The inward movement of the M2 helices that occurs during the pore closure reduces the pore radius at position I233 (I9’) from 6 Å in GLIC open to 4 Å in GLIC LC and likely enables optimal hydrophobic interactions with xenon (Fig 4B). This region of the channel pore has been shown to be critical both for gating and ion transport. Indeed, ordered water molecules (including a pentagon) have been observed at this location in the GLIC open form (Fig 2B) that were shown to be important for ion translocation by allowing the permeant ion to cross hydrophobic barriers [51]. The hydrophobic ring of residues at level I233 (I9’) constitutes a constricted barrier necessary to seal the channel and prevent any ion conduction in the resting state of GLIC [46]. The location of a xenon atom in a functionally critical region of the pore could easily account for the inhibitory effect of xenon on GLIC. Indeed in *Erwinia chrysanthemi* pLGIC (ELIC), another prokaryotic channels of known structure [56], a hydrophobic region of the channel pore bordered by the L9’ and F16’ rings of residues has been shown to be responsible for the binding and the inhibitory effects of bromoform [57], memantine [58] and desflurane [59] (Fig 2B). In addition, the ELIC pore has been shown to bind a xenon atom at the level of the F16’ ring of residues that was used for experimental phasing [56].

#### 3. Xenon interfacial sites mark out a narrow tunnel that links the lipid bilayer to the pore site

In addition, a new interfacial binding site has been probed by xenon, both in GLIC open and LC conformations. It is located between two adjacent subunits in the middle of the membrane-spanning region. It is noteworthy that this site is topologically different from the inter-subunit modulation sites previously characterized in GLIC open form [49] and in ELIC [57]. This interfacial xenon-binding site is both small and hydrophobic which explains why other larger and more polar anesthetics like propofol and desflurane could not bind there. Interestingly, xenon binds here with a very high occupancy (0.9–1) but in two slightly different locations in the open and LC conformations. In GLIC open form, xenon binds between M1 and M2 helices from one subunit and M2 helix from the adjacent subunit in the *inner* part of the TMD while in GLIC LC form, xenon binds between M1 helix from one subunit and M2 and M3 helices from the adjacent subunit in the *outer* part of the TMD. The alternate position of Phe 207 in GLIC open form prevents xenon to bind in the outer interfacial site while the revolving motion of M2 helices which occurs during pore closure suppresses the inner interfacial site. Moreover, these two distinct interfacial xenon-binding sites, termed *inner* and *outer* interfacial sites, are located at the same z-level than the xenon binding site in the pore (Fig 3 and Fig 4B). They mark out a narrow path, membrane-exposed from one end and leading to the pore site on the other end. It is lined by the side chains of residues I236, I232, and Y263 from one subunit and the side chains A234, I208, and F207 from the other subunit.

### Minor xenon binding sites in GLIC ECD and TMD

In the ECD, a xenon-binding site is observed in both GLIC open and LC forms (S1 and S2 Figs) located in a small but highly hydrophobic cavity embedded between the inner and outer β-sheets, whose connecting loops are critical to channel function [42, 60]. Because the ECD region is unchanged between both structures, this site is conserved in both GLIC open and LC structures. A similar cavity also exists in ELIC structure and has been shown to bind bromoform [57]. Interestingly, this region is located close to a binding site for regulatory divalent ions in ELIC [61].

In addition, two minor xenon-binding sites have been observed in the TMD of GLIC (S1 and S2 Figs). The first one is membrane-exposed and is very similar in the open and LC GLIC. It is located in a highly hydrophobic crevice facing the phospholipids, in the middle of the membrane-spanning region. The second one, named minor interfacial site, is present only in the GLIC open form and is located in a small hydrophobic cavity in the middle of the TMD. The distances between the xenon in this site and the xenon in the major inner interfacial site is around 8.5 Å and the two sites are connected through a narrow tunnel.

## Discussion

Structural studies on xenon-binding properties of GLIC can shed light and improve our understanding of how it modulates its function. In general, xenon binds through weak but specific interactions to pre-existing internal cavities or accessible pockets lined by hydrophobic residues [21, 34, 62]. Xenon binding within cavities, like other anesthetics, modifies at the molecular level the dynamics of their targets, increasing their stability and shifting equilibria between conformational states, like close and open states in the case of channel receptors [63–65]. Functional assays in presence of inert gases (xenon and nitrous oxide) have revealed two mechanisms of inhibition in globular proteins. In urate oxidase for example, xenon inhibited enzymatic activity though an indirect and non-competitive mechanism by binding to an allosteric internal cavity located close to the active site. Xenon induced an expansion of the volume of this hydrophobic cavity, which increased with the applied gas pressure, leading to an increase of the rigidity of the cavity and of the flexibility to the neighboring active site [36, 37, 66]. On the other hand, in elastase and tissue-type plasminogen activator (t-PA), xenon inhibited enzymatic activity through a direct and competitive mechanism by binding directly in the active site of the two enzymes [36, 67].

Here we present a structure where xenon is bound to an integral transmembrane receptor, GLIC. This is an important step for studying the molecular basis of xenon-binding and modulation of its physiological transmembrane receptor targets because i) GLIC is inhibited by clinical concentrations of xenon [41] and ii) its crystallographic structure is known in different allosteric states, including its opened and LC (inactive) forms. The TMD of GLIC LC-form adopts a conformation that is very similar to that of the resting state. Structurally, it is striking that the description of the LC form matches very well a functional intermediate between the resting and activated that is “agonist-bound” but inactive (closed pore), that has been postulated by several authors [68]. Furthermore, several GLIC X-ray structures have been solved in complex with both injected and inhaled general anesthetics like propofol, desflurane [47], bromoform [49], as well as in complex with ethanol [49, 69, 70]. Xenon-binding sites can thus be compared with other allosteric modulator binding sites in order to decipher which binding sites are specific to xenon and which are shared with other modulators.

Xenon major binding sites are located in three distinct regions in GLIC, all in the trans-membrane domain (TMD): i) in an intra-subunit cavity, ii) at the interface between adjacent subunits, iii) in the pore (this latter site being specific to the LC form). Xenon binds to pre-existing cavities that were previously shown to bind other volatile or intravenous GAs, suggesting that some modulation sites are common to all GAs, including the gaseous ones. However, other sites appear specific to xenon, suggesting that its particular size and physico-chemical properties allow xenon to target additional modulations sites. Interestingly, all major xenon binding sites differ in the two allosteric conformations, consistent with the hypothesis that anesthetics would bind in regions that show large conformational modifications during the transition between the opened and closed states [71].

In GLIC open form, the intra-subunit binding site can be considered as a common anesthetic binding site, since it can bind not only xenon, but other anesthetics like propofol, desflurane or bromoform. The intra-subunit site has formerly been described as an inhibitory site for GAs in GLIC [47]. A recent study on the *Torpedo marmorata* nAChR using a photoreactive propofol analogue mapped exactly the same intra-subunit propofol binding site suggesting that this cavity of GLIC is a strong candidate for the nAChR allosteric inhibitory site [72]. However, the molecular mechanism of inhibition induced by GAs upon binding to this intra-subunit site remains an open question, since existing GLIC X-ray structures of the receptor bound to GAs all displayed an open conformation while GAs should promote a closed state of the receptor. The xenon-bound GLIC structure in its LC conformation provides now the picture of the intra-subunit cavity in an agonist-bound but inactive (closed) conformation of the receptor. Interestingly, the conformational modification between open and LC GLIC leads to an important constriction of this cavity: while xenon can occupy three distinct positions in the GLIC open form it is restricted to one single position in GLIC LC form. This observation suggests that access of larger GAs to the intra-subunit cavity would also be prohibited in GLIC LC. At this stage it would be difficult to suggest a mechanism that would account for the allosteric inhibition only through the *intra-subunit* cavity binding. On the other hand, one might hypothesize that GAs binding to the intra-subunit cavity may inhibit the channel through stabilizing a desensitized form of the channel, where this cavity might be re-modeled; however, the X-ray structure of the desensitized form of GLIC is currently unknown. Further investigations including a crystal structure of GAs-bound GLIC in a desensitized form will be necessary in order to formally clarify this point.

The novel xenon interfacial binding sites in GLIC described in this paper are distinct from those of GABA_A_ and Glycine receptors whose interfacial GAs binding site is associated to potentiation [73]. The inner (in open conformation) and outer (in LC conformation) interfacial sites are located in two different positions due to the revolving motion of M2 helices, which occurs during pore closure. Several observations suggest that xenon could access its protein sites from the plasma membrane. Indeed, xenon's high polarizability increases its affinity for hydrophobic environments so that under physiological conditions a fraction of xenon atoms probably permeates into the membrane. Simulations studies have suggested that xenon is localized in the hydrophobic core of the bilayer [74–76]. This lead us to suggest that the outer and inner interfacial xenon binding sites, located at the level of the membrane spanning-region, could mark out a convenient path for xenon atoms to target the pore inhibitory site. Even if this path is narrow, fluctuations of individual side chains might allow xenon to migrate from one interfacial binding site to the other and to reach the pore site [29, 31].

The xenon atom located at the center of the pore on the five-fold non-crystallographic symmetry axis is of particular interest. This site is specific to the GLIC LC form, not the open form. The Xe atom is surrounded by the side chains of five Ile 233 (I9’) residues at a distance of 4.4 Å. This distance would increase to 5.6 Å in the GLIC open form, too large for an efficient binding. Indeed, optimal distances between Xe atom and carbon atom of aliphatic side chain range between 3.5 Å and 4.8 Å [21]. Instead, in GLIC open form, the corresponding xenon pore binding site is occupied by a pentagon of ordered water molecules that are known to be important for the ion to cross hydrophobic constriction barriers [51]. The binding of xenon to this site might alter locally the water organization in a way that would facilitate the hydrophobic gating at the level of the Ile 233 ring of residues, leading to its inhibitory effect. Prior to this work, two xenon binding sites have been found in the pore of ELIC, in its non-conducting conformation, one in a hydrophobic cavity inside the pore and the second on the extracellular side of the pore [56]. In that case however, xenon was only used for its phasing properties. More recently, bromoform has been shown to bind to a similar hydrophobic region in the channel pore of ELIC and a site-directed mutagenesis study of the L9’ and F16’ rings of pore-lining residues revealed that bromoform binding to the pore accounted for its inhibitory effects [57, 59]. Moreover, molecular dynamics studies predicted that GAs could block the GLIC pore [77] and that the pore binding site might constitute an inhibitory site shared with other pLGICs.

It is important to note that the pore site should not be considered as the unique xenon modulation site in GLIC. None of our findings preclude the possibility that, in addition to its direct action on the pore site of GLIC, xenon affects channel functions through other ways, such as membrane-exerted mechanisms caused by its accumulation in the lipid bilayer, and stabilisation of other inactive state(s) by binding to intra and inter-subunits cavities. Likewise, the binding of Xenon by an heteropentamer instead of a homopentamer like GLIC might reveal additional sites.

Contrary to volatile and intravenous anesthetics that mainly target GABA_A_ receptor, xenon barely affects it, but mainly inhibits NMDA receptor [5, 13, 17]. However, inhibition by xenon of excitatory pLGIC receptor like nACh receptor has also been reported [15, 16, 19], and it has been suggested that inhibition of nACh receptors by xenon could be involved in hypnosis mechanism [78] but not in analgesia [5]. Since GLIC displays a pharmacological profile that, to some extent, resembles that of nACh receptor, our work might be relevant for a molecular understanding of the inhibition of nAChR by xenon.

## Conclusion

X-ray crystallography under pressurized xenon successfully allowed to identify xenon-binding sites in the GLIC receptor and to propose a molecular mechanism of modulation. Indeed, solving the structures of the xenon-bound GLIC receptor in both active and inactive conformations revealed that modulation sites are profoundly remodelled from one conformation to the other. Further studies will be needed to assess if this observation can be extended to other transmembrane receptors that are modulated by xenon.

## Supporting Information

S1 Fig Minor xenon-binding sites in GLIC open (left) and LC conformations (right). Top panels: the receptors are viewed from the side with the two front subunits removed to allow a better visualization of the xenon-binding sites. Bottom panels: the TMD are viewed from the top. Regions of the TMD that differs between the open and LC GLIC conformations are highlighted in yellow, other regions of the TMD are coloured in grey; the ECD is coloured in cyan. The anomalous (orange) and 2mFo-dFc averaged Fourier difference (blue) maps surrounding the xenon minor binding sites are shown by mesh contoured at 3.0 σ and 1.0 σ, respectively. Xenon atoms are shown by red van der Waals spheres in its minor binding sites and by pink spheres in its major binding sites for comparison.
(TIF)

S2 Fig Detailed view of the minor xenon-binding sites. Top panels show the ECD xenon-binding site in GLIC and the equivalent bromoform binding site in ELIC. The bottom-left panel represents the minor interfacial xenon-binding site that is observed in GLIC open form. The bottom-right panel represents the membrane exposed xenon-binding site as well as a putative phospholipid that binds next to it (grey). Receptors are shown as cartoons while sticks (blue) are used to highlight side chains of residues neighbouring xenon-binding sites. Xenon atoms represented by van der Waals spheres (magenta). Xenon-binding cavities in GLIC are delimited by a transparent white surface.
(TIF)
