# Supplementary figures and images for: Structural Basis for Xenon Inhibition in a Cationic Pentameric Ligand-Gated Ion Channel
Source: PLoS One. 2016 Feb 24;11(2):e0149795. doi: 10.1371/journal.pone.0149795 (PMC4765991; doi:10.1371/journal.pone.0149795)

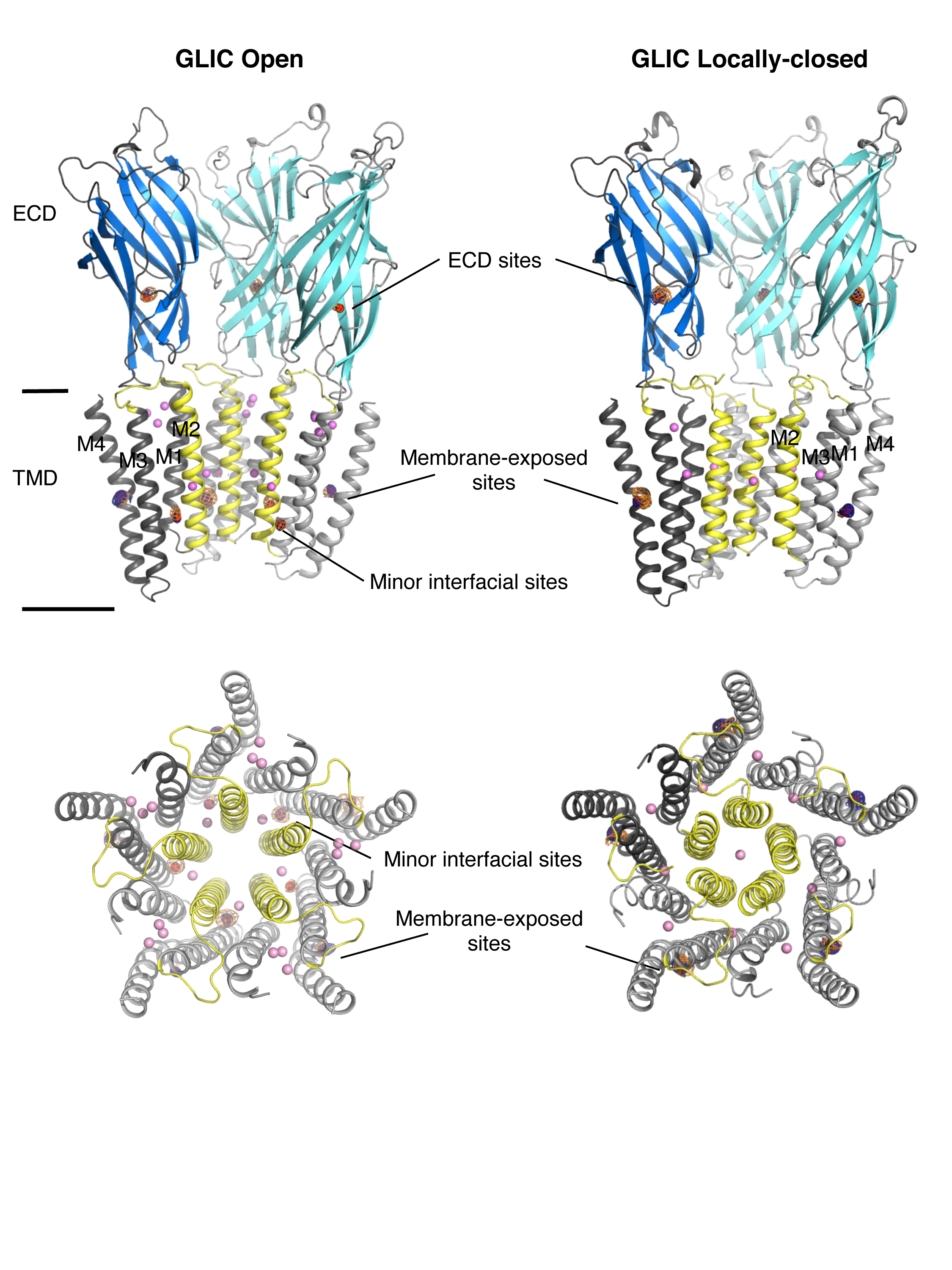

Supplement: S1 Fig — Top panels: the receptors are viewed from the side with the two front subunits removed to allow a better visualization of the xenon-binding sites. Bottom panels: the TMD are viewed from the top. Regions of the TMD that differs between the open and LC GLIC conformations are highlighted in yellow, other regions of the TMD are coloured in grey; the ECD is coloured in cyan. The anomalous (orange) and 2mFo-dFc averaged Fourier difference (blue) maps surrounding the xenon minor binding sites are shown by mesh contoured at 3.0 σ and 1.0 σ, respectively. Xenon atoms are shown by red van der Waals spheres in its minor binding sites and by pink spheres in its major binding sites for comparison. (TIF) [file pone.0149795.s001.tif]

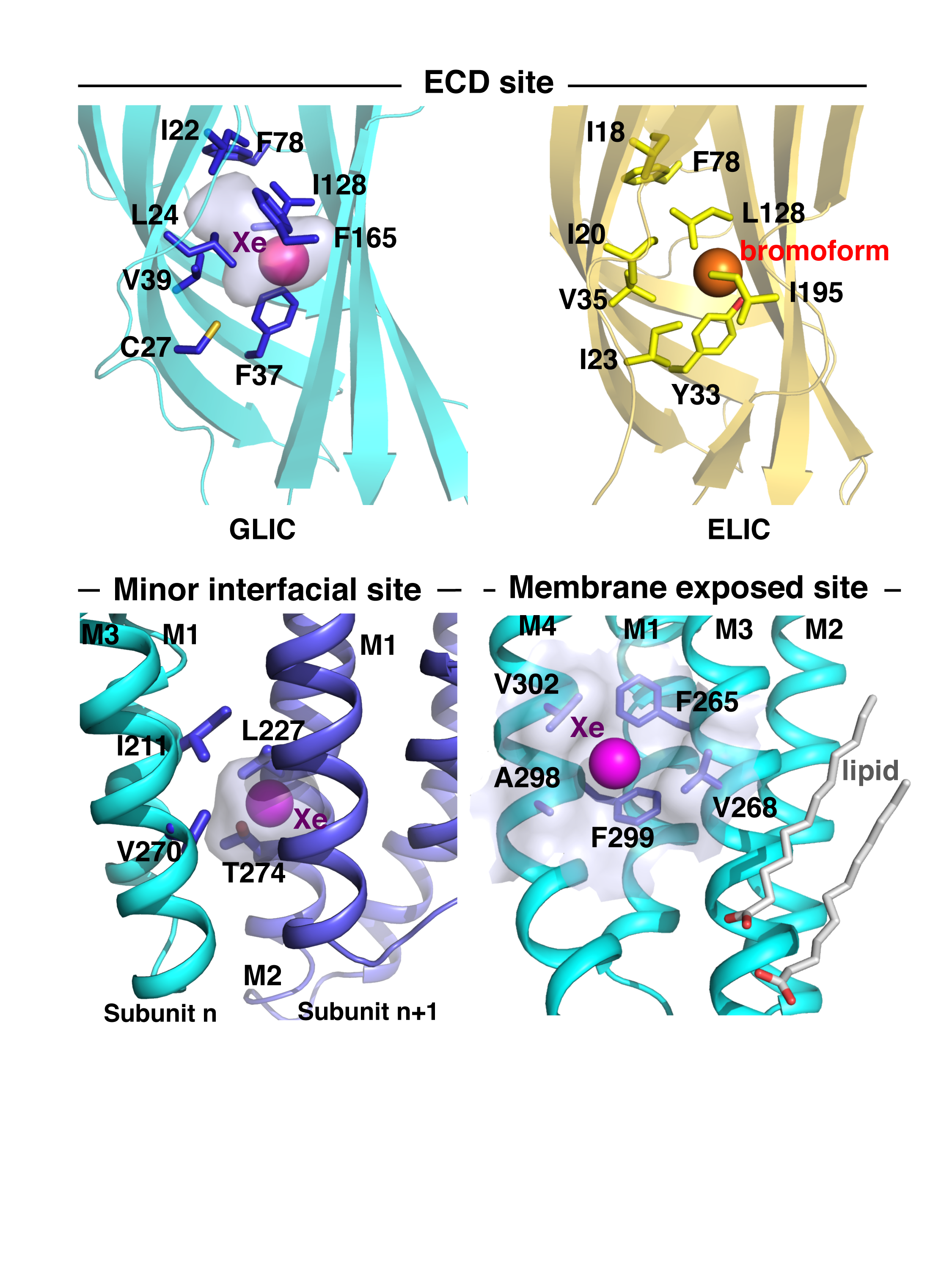

Supplement: S2 Fig — Top panels show the ECD xenon-binding site in GLIC and the equivalent bromoform binding site in ELIC. The bottom-left panel represents the minor interfacial xenon-binding site that is observed in GLIC open form. The bottom-right panel represents the membrane exposed xenon-binding site as well as a putative phospholipid that binds next to it (grey). Receptors are shown as cartoons while sticks (blue) are used to highlight side chains of residues neighbouring xenon-binding sites. Xenon atoms represented by van der Waals spheres (magenta). Xenon-binding cavities in GLIC are delimited by a transparent white surface. (TIF) [file pone.0149795.s002.tif]
